# Development of ovine hepatic organoids: a powerful *in vitro* platform to reduce the number of experimental animals used in metabolism and nutrition assays

**DOI:** 10.3389/fvets.2026.1817725

**Published:** 2026-05-11

**Authors:** María-Cristina González-Montero, Miguel Criado, Mercedes Alonso, Nuria Santos, Daniel Gutiérrez-Expósito, F. Javier Giráldez, Rafael Balaña-Fouce, Sonia Andrés, Carlos García-Estrada

**Affiliations:** 1 Departamento de Ciencias Biomédicas, Facultad de Veterinaria, Universidad de León, León, Spain; 2 Instituto de Ganadería de Montaña, CSIC-Universidad de León, León, Spain; 3 Instituto de Biomedicina (IBIOMED), Universidad de León, León, Spain; 4 Instituto de Investigación Biosanitaria de León (IBIOLEÓN), León, Spain

**Keywords:** 3D cultures, 3Rs, alternative methods, betaine, liver, methionine, organoids, sheep

## Abstract

**Background:**

The liver coordinates metabolic processes that determine growth, health, and production efficiency in livestock species. In ruminants, hepatic metabolism is adapted to support glucose homeostasis, lipid partitioning, and nitrogen utilization during ruminal fermentation. Therefore, predictive experimental models are essential for studying nutrient-mediated regulation of liver function. However, conventional two-dimensional hepatocyte cultures fail to reproduce the structural organization and functional complexity of native liver tissue, limiting their translational value.

**Methods:**

Hepatic organoids were developed from the progenitor cells of the liver of a male Assaf lamb, and were characterized by histological and immunofluorescence analyses, followed by transcriptomic profiling using RNA-seq and functional enrichment analysis. The metabolic response was assessed by adding DL-methionine and/or betaine and analyzing, by RT-qPCR, the expression of genes encoding key metabolic enzymes (e.g., *CPT1A* and *PDH1*) and ribosomal proteins representative of cellular proliferation (e.g., *RPL22L1*).

**Results:**

Hepatic organoids formed spherical epithelial structures with defined apical–basal polarity, intact tight junctions, albumin expression, and intracellular glycogen accumulation. Comparative transcriptomic analysis between organoids and native liver tissue supported the conservation of core cellular programs, although differential expression and functional enrichment analyses demonstrated that hepatic organoids exhibit a transcriptional profile biased toward cell proliferation, protein synthesis, and structural remodeling, while genes associated with mature liver metabolic functions, immune responses, and systemic homeostasis were relatively downregulated. Combined treatment with DL-methionine and betaine significantly increased the expression of *CPT1A* (associated with mitochondrial beta-oxidation) and *RPL22L1* genes, indicating a synergistic metabolic response of both compounds.

**Discussion:**

Ovine hepatic organoids reproduced structural and cellular features of liver tissue, supporting their relevance as an *in vitro* metabolic model for ruminants. Transcriptomic comparisons revealed incomplete functional maturation relative to adult liver, which is consistent with a progenitor-like hepatic state commonly observed in organoid systems. Nevertheless, the preserved metabolic response of organoids highlights their value for studying nutrient–gene interactions and evaluating dietary interventions prior to *in vivo* experimentation. This work represents the first report of sheep hepatic organoids and establishes a foundation for their use as predictive *in vitro* platforms to study hepatic metabolism, nutrient–gene interactions, and feed additive strategies in ruminant livestock.

## 1 Introduction

The liver is a central metabolic organ that coordinates diverse physiological processes critical for organismal homeostasis. It orchestrates amino acid and nitrogen metabolism, lipid synthesis and oxidation, carbohydrate processing, xenobiotic detoxification, and nutrient sensing, collectively influencing growth, health, and production traits in mammals (1). These integrated functions allow the liver to modulate systemic energy balance, respond to nutritional fluctuations, and regulate biosynthetic and catabolic pathways. This is especially important in animal production, as imbalances in nutrient supply—whether deficiencies or excesses—can lower milk or meat production efficiency (2, 3).

Ruminant species, such as sheep and cattle, exhibit distinct metabolic characteristics that differentiate them from monogastric animals. Due to ruminal fermentation, very little glucose is absorbed directly from the intestine, and propionate—one of the main volatile fatty acids produced in the rumen—serves as the primary substrate for hepatic gluconeogenesis, providing most of the glucose required for energy and lactation (4). In order to maximize gluconeogenesis, the activity of the enzyme pyruvate dehydrogenase is limited in the liver of ruminants, thus regulating the oxidation of glucose precursors (e.g., pyruvate, generated by glycolysis) before entering the tricarboxylic acid cycle. Mitochondrial fatty acid oxidation in the liver of ruminants is also necessary for energy production and metabolic adaptation. Additionally, the rumen microbiota plays a central role in nitrogen and amino acid metabolism, converting dietary protein into microbial protein while recycling nitrogen, which is essential for production efficiency and minimizing nitrogen losses to the environment (5). These unique hepatic and microbial metabolic pathways allow ruminants to efficiently extract energy and nutrients from fibrous diets, regulate lipid partitioning, and maintain growth and product quality under diverse nutritional conditions (6).

Given that hepatic metabolism is a key determinant of animal production traits, including growth efficiency and nutrient utilization, the development of robust experimental systems for advancing both basic biology and translational applications in livestock species is essential. Historically, two-dimensional (2D) hepatocyte cultures and conventional cell lines have served as *in vitro* models for hepatic research. However, these systems fail to reproduce the complex cytoarchitecture of the organ, lack spatial gradients of nutrients and oxygen, exhibit abnormal liver-specific functions, do not recapitulate three-dimensional (3D) cell–matrix and cell–cell interactions, and provide limited predictive power for *in vivo* metabolic outcomes (7). As a result, primary hepatocytes cultured in conventional 2D monolayers undergo extensive transcriptomic and proteomic changes relative to intact liver tissue, including altered gene expression, which can limit their physiological relevance as models of hepatic function (8). To circumvent these problems, 3D hepatic organoids—multicellular, self-organizing structures derived from primary tissue, progenitor cells, or pluripotent stem cells—have emerged as advanced *in vitro* platforms that recapitulate key features of native hepatic tissue, including polarized cell organization, bile duct-like structures, activity of drug-metabolizing enzymes (e.g., CYP450), and functional outputs such as albumin secretion and metabolic enzyme expression, enabling more accurate modeling of liver function (9, 10). These attributes make organoids powerful tools for disease modeling, drug toxicology assessment, and mechanistic studies of hepatic metabolism (10, 11).

In biomedical research, liver organoids have been applied to study human liver development, disease pathogenesis, and therapeutic responses, often outperforming traditional *in vitro* and animal models by better preserving cellular heterogeneity and functional fidelity (12). Despite these advances, organoid technology has been largely underutilized in livestock research, where predictive *in vitro* models are critically needed to investigate nutrient metabolism, metabolic disorders, and species-specific physiology in animals such as sheep or cow (13). In fact, although *in vivo* studies are essential to elucidate aspects of ruminant hepatic metabolism, they are resource intensive and ethically constrained, underscoring the need for representative *in vitro* systems (14). To address this gap, we report here the establishment and characterization of ovine hepatic organoids and perform comparative transcriptomic profiling (RNA-seq) between organoids and native ovine liver tissue (primary tissue of origin) to evaluate the extent to which organoids recapitulate *in vivo* expression patterns. Additionally, we evaluate metabolic responsiveness by quantifying the expression of selected metabolic genes via RT-qPCR following supplementation with DL-methionine and/or betaine. To the best of our knowledge, this is the first report on sheep hepatic organoids and the data presented in this article demonstrate the potential of 3D ovine liver organoids as predictive *in vitro* models for studying hepatic metabolism in livestock, providing a foundation for mechanistic studies of nutrient-mediated regulation and trait-associated metabolic outcomes.

## 2 Materials and methods

### 2.1 Generation and culture of sheep hepatic organoids

Sheep hepatic organoids were obtained from hepatic progenitor cells isolated from a male Assaf lamb (45 days of age). All animal experiments complied with Spanish and European Union regulations (RD 53/2013 and 2010/63/EU) and were approved by the CSIC Animal Experimentation Committee and the relevant authority (protocol number 100102/2021-6, authorized on 31 January 2022).

The protocol followed to develop the hepatic organoids was similar to that reported for mouse hepatic organoids (15). Briefly, a 3-g piece of the central area of a liver lobule was collected post-mortem from male lamb, minced, and digested using a tissue dissociation protocol. The resulting cell suspension was filtered, pelleted, and embedded in Matrigel® matrix (Corning®, Discovery Labware Inc., Bedford, MA, United States) in 24-well plates. Domes were overlaid with HepatiCult™ Mouse Organoid Growth Medium (StemCell Technologies™, Vancouver, BC, Canada) and cultured at 37 °C with 5% CO₂, with medium changes every 2–3 days. Organoids were subcultured weekly by disrupting the matrix in ice-cold Advanced DMEM/F-12 (Gibco, Life Technologies Limited, Paisley, UK) by gentle pipetting and re-plating the pelleted cells following the same procedure. Organoid growth was monitored over a 7-day period by capturing images from 15 randomly chosen organoids per time point, whose diameters were measured with a Nikon Eclipse Ti2 microscope (Nikon Instruments Inc., Tokyo, Japan) coupled with an Imaging Source DMK 33UX174 camera (The Imaging Source Europe GmbH, Bremen, Germany).

After three passages, organoids from two wells, each containing approximately 200 structures, were harvested for long-term preservation. The extracellular matrix was dissociated using 1 mL of CryoStor® CS10 (StemCell Technologies™, Vancouver, BC, Canada), and the resulting organoid suspension was transferred into cryovials for storage in liquid nitrogen until further use.

### 2.2 Histochemical and immunofluorescence staining

For histochemical and immunofluorescence characterization, 7-day-old organoids were fixed in 4% methanol-free paraformaldehyde (Thermo Fisher Scientific, Waltham, MA, United States) prepared in phosphate-buffered saline (PBS) for 30 min at room temperature (RT). Following fixation, organoids were released from Matrigel® domes by incubation in ice-cold PBS and subsequently washed twice in PBS. To minimize mechanical damage, all manipulations were performed using wide-bore, trimmed pipette tips.

For histological processing, organoids were embedded in agarose using a protocol adapted from previously published methods (16). Briefly, 2% high-purity agarose (Cat. No. 4757, Biotools, Madrid, Spain) was dissolved in PBS and allowed to cool to approximately 60 °C in a 50 mL conical tube. Organoids were then resuspended in the agarose, which was solidified on ice. After solidification, agarose blocks containing the organoids were removed and post-fixed in formaldehyde, followed by routine paraffin embedding. Paraffin sections of 3 μm thickness were obtained and mounted on poly-L-lysine–coated slides (SuperFrost Plus, Thermo Fisher Scientific, Waltham, MA, United States). Sections were stained with hematoxylin and eosin (H&E) or with Alcian Blue (pH 2.5) followed by Periodic Acid–Schiff (PAS) to visualize glycogen as well as acidic and neutral glycoconjugates, including mucins and glycoproteins.

For whole-mount immunofluorescence, organoids were permeabilized and blocked simultaneously by incubation in PBS containing 0.1% Triton™ X-100 (Sigma-Aldrich, St. Louis, MO, United States) and 3% bovine serum albumin (BSA) (Roche Diagnostics, Mannheim, Germany) for 1 h at RT, under gentle agitation on a plate shaker to maintain organoids in suspension. Organoids were then incubated with fluorophore-conjugated antibodies as previously described (15). The following antibodies were used: a rat anti–ZO-1 (Zonula Occludens 1) Alexa Fluor 594–conjugated antibody (clone R40.76, sc-33725 AF594, Santa Cruz Biotechnology, Dallas, TX, United States) and a rabbit monoclonal anti-albumin Alexa Fluor 750–conjugated antibody (NBP3-08917AF750, Novus Biologicals, Centennial, CO, United States). Both were diluted 1:50 in PBS supplemented with 3% BSA and 0.1% Triton™ X-100, and incubated for 2 h at room temperature with gentle agitation. Organoids were then washed five times with PBS containing 0.05% Tween-20 (PBS-T) and mounted in Fluoroshield™ mounting medium with DAPI (Sigma-Aldrich, St. Louis, MO, United States). Whole mounts were placed onto poly-L-lysine–coated slides using #1 coverslips as spacers to prevent sample compression.

Wide-field epifluorescence imaging was performed using a Nikon Eclipse Ni-E microscope (Nikon, Tokyo, Japan) equipped with a Prime BSI scientific CMOS camera (Photometrics®, Scottsdale, AZ, United States), and multi-channel images were merged using NIS-Elements Advanced Research software (v6.20.00, Nikon). Confocal imaging was conducted using a Zeiss LSM 800 confocal microscope (Observer Z1, Carl Zeiss, Oberkochen, Germany). For three-dimensional visualization, confocal Z-stacks were acquired at 0.21 μm intervals. Image restoration was performed via 3D deconvolution using a constrained iterative algorithm within ZEN Blue Edition (v2.6.76.00000; Carl Zeiss). The deconvolution pipeline utilized GPU acceleration, and a calculated point spread function (PSF), applying automatic normalization and first-order regularization. Processing was based on Poisson likelihood, using mean intensity as the initial estimate. To ensure data integrity and prevent over-processing, the algorithm was limited to 7 iterations per channel with an auto-stop convergence threshold of 0.1%.

### 2.3 RNA-seq analysis

Transcriptome-wide RNA-seq analysis was performed using samples comprising five pieces of hepatic tissue (~1 cm) from a male Assaf lamb (45 days of age), and sheep hepatic organoids collected from five different wells. Samples were preserved in RNAlater® (Fisher Scientific, Waltham, MA, United States) and stored at −80 °C prior to RNA extraction. RNA extraction, library preparation and sequencing on an Illumina NovaSeq X platform (2 × 150 bp, paired-end) were carried out by Seqplexing Genetest (Valencia, Spain) as previously described (15). Post-mapping quality control to quantify reads mapped to each gene indicated over 95% of reads successfully aligned for most samples. Gene expression was quantified using HTSeq-count, normalized with DESeq2, and differential expression was assessed with False Discovery Rate correction according to the Benjamini–Hochberg method. Data visualization was performed in R (version 4.4.2) using packages such as ggplot2 (version 3.5.1) and pheatmap (version 1.0.12) to generate heatmaps, Volcano plots, Principal Component Analysis (PCA) plots, and dispersion curves. Differentially expressed genes were classified as upregulated or downregulated based on a Log2FC < −1 or >1, respectively, and a *q*-value < 0.1. Gene identifiers were standardized to ENTREZ Gene IDs, achieving a conversion success rate exceeding 99%.

Functional enrichment analyses, including KEGG pathways and gene ontology categories (biological process, cellular component and molecular function) were conducted using DAVID web server (v2024q4) (17, 18) of the NIH (DAVID Functional Annotation Bioinformatics Microarray Analysis).

### 2.4 Ovine hepatic organoid response to methyl donor supplementation

Organoids (50–100 per well) were grown in two different 24-well plates for 4 days, and the most homogeneous wells (12 wells per plate) were selected to apply the experimental treatments as follows: 2 plates (biological replicates) × 4 treatments × 3 technical replicates per plate and treatment. Then, experimental treatments [750 μL of 0.02 mM DL-methionine, betaine, or DL-methionine + betaine solutions diluted in monolayer medium (Hepaticult™)] were added to the domes of each well. After 24 h of incubation, hepatic organoids were collected for total RNA extraction using the GeneMATRIX Universal RNA Purification Kit (EURx Ltd., Gdansk, Poland) following manufacturer’s instructions. The RNA quantity was measured using the QuantiFluor® RNA System and a QuantusTM Fluorometer (Promega) and the RNA integrity number determined using Bioanalyzer 2100 (Agilent Technologies, Santa Clara, CA, United States). Total RNA was reversed-transcribed to cDNA using the Invitrogen™ SuperScript™ VILO™ Master Mix, according to the manufacturer’s instructions. The cDNA was used as a template for quantitative real-time PCR analysis (RT-qPCR). Finally, the expression of genes encoding mitochondrial β-oxidation of fatty acids (e.g., *Ovis aries* carnitine palmitoyltransferase 1A, *CPT1A*), mitochondrial glucose oxidation and cellular respiration (e.g., pyruvate dehydrogenase (lipoamide) alpha 1, *PDHA1*) and cellular proliferation (e.g., ribosomal protein L22 like 1, *RPL22L1*) was assessed by RT-qPCR using the pairs of primers described in Table 1. The Ct values were normalized according to the expression of the *B2M* gene (reference housekeeping gene encoding beta-2-microglobulin) and the results expressed as the efficiency-corrected target quantity (N_0_) calculated by the LinRegPCR program.

**Table 1:** Primers used for RT-qPCR amplification and the study gene of expression in ovine hepatic organoids.

Gene	NCBI reference	Forward primer	Reverse primer	Product size
*CPT1A*	NM_001009414.1	TGAAAAGGCAGCGTTCTTCG	AAACCACCTGTCGAAACACC	127 bp
*PDHA1*	XM_012140344.2	TCATTGAAGGTTCGCAGCTG	TTGCAAAATGACGGGATGCC	133 bp
*RPL22L1*	XM_004003165	TTCGTGATTGGCTTCGTGTG	GCAAGCAAAGCCCTATGAAGG	132 bp
*B2M*	XM_060418694.1	TTCATTGTGCCTGCCTTTCC	TGCAAAACACCCTGACCAAG	138 bp

### 2.5 Statistical analysis

Organoid diameter was analyzed using a linear mixed model (SAS PROC MIXED, SAS Institute Inc.). Individual well and well by-time interaction were treated as random effects to account for the hierarchical structure and organoid subsampling. To accommodate the observed increase in diameter variance over time, a heterogeneous variance structure was specified using the GROUP option. The Satterthwaite method was used to estimate denominator degrees of freedom.

The efficiency-corrected target quantity (N_0_) values obtained for each gene by RT-qPCR were analyzed also using a Linear Mixed Model (PROC MIXED) in SAS, with treatment (control, DL-methionine, betaine or DL-methionine+betaine) as a fixed effect and Plate as a random block. To account for the hierarchical structure, the Plate-by-Treatment interaction was included as the error term for treatment effects, while the three wells per group served as technical replicates. Degrees of freedom were adjusted via the Kenward-Roger method. In both models, significant differences between means were identified using Tukey’s HSD test at a significance level of *p* < 0.05. Model assumptions were verified by applying the Shapiro–Wilk test to studentized residuals.

## 3 Results

### 3.1 Development and characterization of sheep hepatic organoids

**Figure 1:**
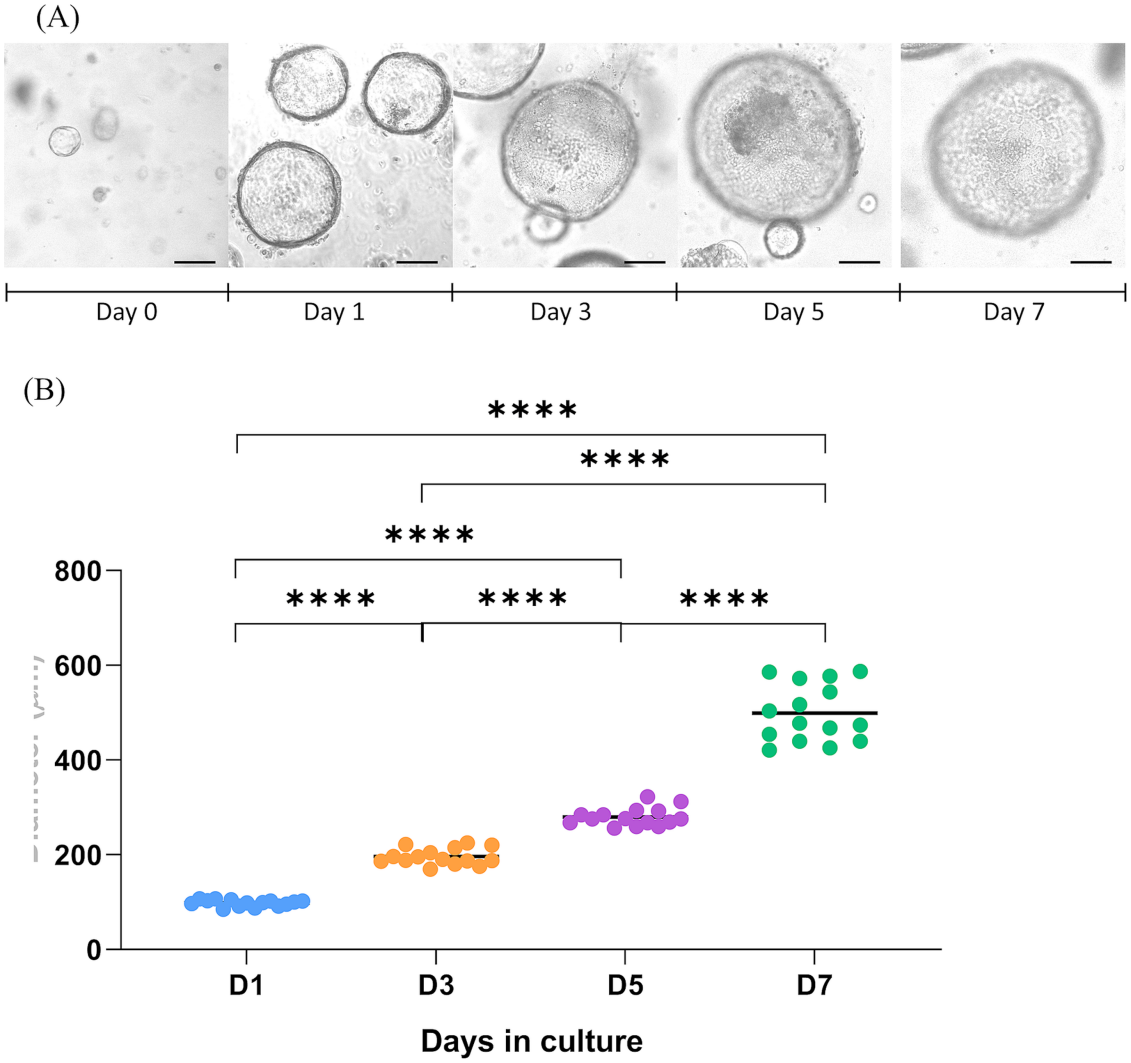
Growth of sheep hepatic organoids along the culture time. **(A)** Representative brightfield microscopy images of sheep hepatic organoids at different time-points. Day 0 refers to the time point of seeding the cell pellet, resuspended in Matrigel^®^ Matrix Basement Membrane Phenol-Red Free (Corning^®^, Discovery Labware Inc., Bedford, MA, United States), after cell passage. Pictures were taken with a Nikon Eclipse Ti2 microscope (Nikon Instruments Inc., Tokyo, Japan) coupled with an Imaging Source DMK 33UX174 camera (The Imaging Source Europe GmbH, Bremen, Germany). Scale bars = 100 μm. **(B)** Graphical representation of the sheep hepatic organoid growth over a 7-day period (D1–D7). Diameter (μm) was measured for 15 organoids at each time point. ****p ≤ 0.0001.

Sheep hepatic organoids showed a round shape throughout the culture period (Figure 1A), exhibited rapid growth during the first 7 days in culture, with significant differences in size relative to earlier time points (Figure 1B), and began to disaggregate on day 8–9 if not passaged. After three passages, sheep hepatic organoids were cryopreserved in liquid nitrogen and revived after 5 months, with no morphological differences relative to the parental organoids.

**Figure 2:**
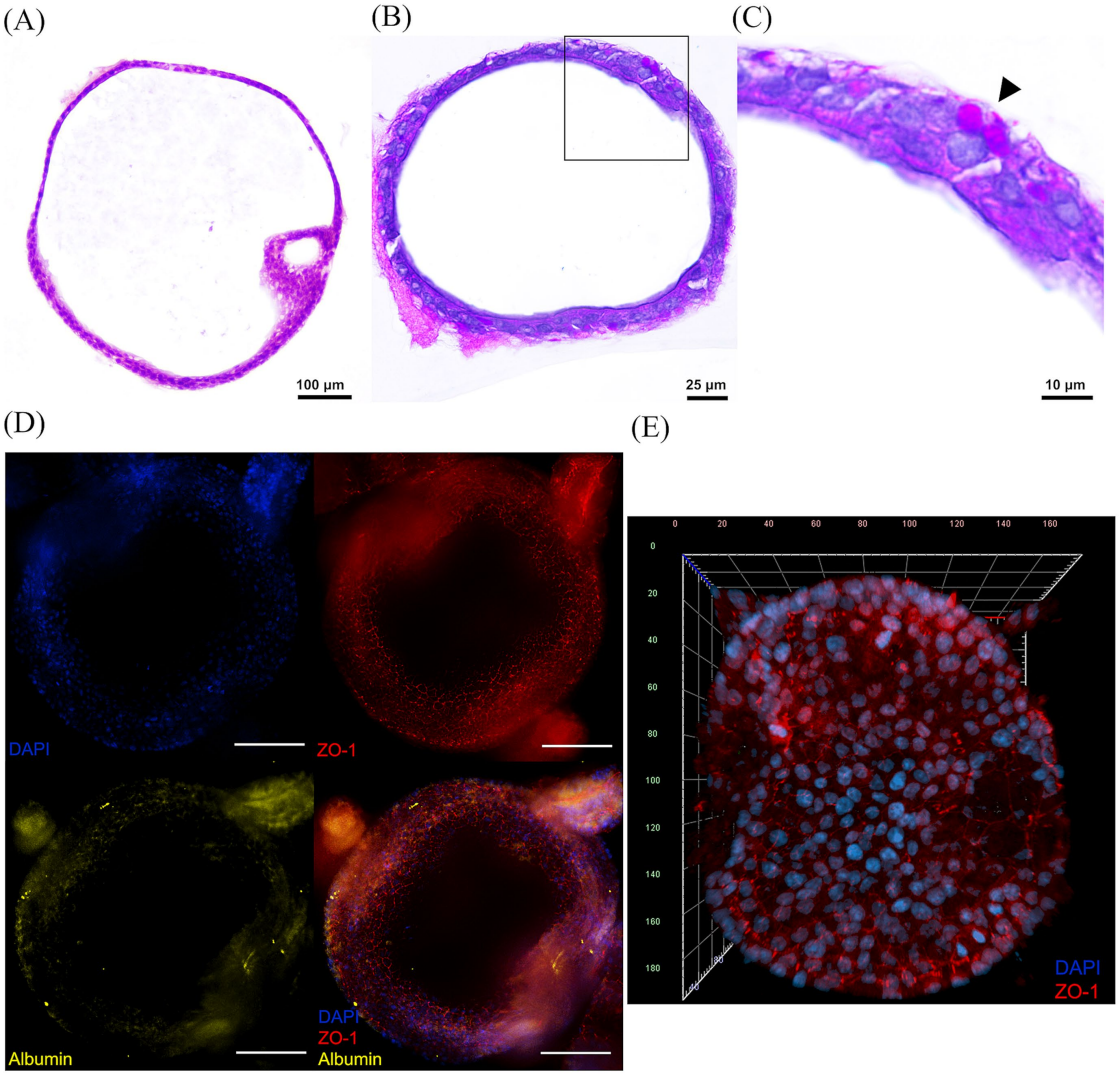
Microscopic analysis of sheep hepatic organoid sections after 7 days of *in vitro* culture. **(A)** H&E staining revealed that the organoids are composed of polygonal cells surrounding a closed central lumen. The cells primarily form a monolayer, though partial stratification can occur in larger organoids, as seen in the lower half of the image. **(B)** Alcian Blue combined with Periodic Acid Schiff’s (PAS) staining revealing cytoplasmic PAS-positive vacuoles in the basal side on several cells, indicative of intracellular glycogen storage. **(C)** Inset of **(B)** (boxed region in panel **B**) highlighting PAS-positive vacuoles (arrowhead). **(D)** Wide-field epifluorescence micrographs of an organoid stained with 4′,6-diamidino-2-phenylindole (DAPI, nuclei), Zonula Occludens 1 (ZO-1, tight junctions) and albumin. The merged image shows colocalization of the three elements; scale bars = 100 μm. Images from panels **(A–D)** were taken with a Nikon Eclipse Ni-E microscope (Nikon, Tokyo, Japan) equipped with a Prime BSI Scientific CMOS camera (Photometrics^®^ Prime BSI™, Scottsdale, AZ, United States). **(E)** Three-dimensional confocal reconstruction of an organoid stained with DAPI and labeled against ZO-1, demonstrating apical-in polarity and basally localized nuclei; scale bar in μm. Confocal imaging was performed on a Zeiss LSM 800 (Observer Z1, Carl Zeiss, Oberkochen, Germany).

Staining with H&E revealed that organoids formed closed, spherical epithelial structures, typically consisting of a continuous monolayer of polygonal cells enclosing a central lumen, although some organoids showed partial stratification. These cells exhibited cuboidal to oval morphology, basophilic nuclei, and eosinophilic cytoplasm (Figure 2A). Staining with PAS combined with Alcian blue revealed the presence of intracytoplasmic vacuoles indicative of glycogen accumulation (Figures 2B,C).

Immunofluorescence analysis was conducted to assess both apical polarity, tight junction integrity and hepatic function (Figure 2D). Staining with DAPI revealed that nuclei are located basally, narrow ZO-1–positive junctions were observed between adjacent cells, toward the apical side and albumin expression was detected in most cells at varying intensities. Finally, confocal microscopy was employed for three-dimensional reconstruction of the organoids allowing a more precise structural characterization, which confirmed an apical-in polarity (Figure 2E).

### 3.2 Comparison of transcriptional profiles between sheep hepatic organoids and liver tissue

**Figure 3:**
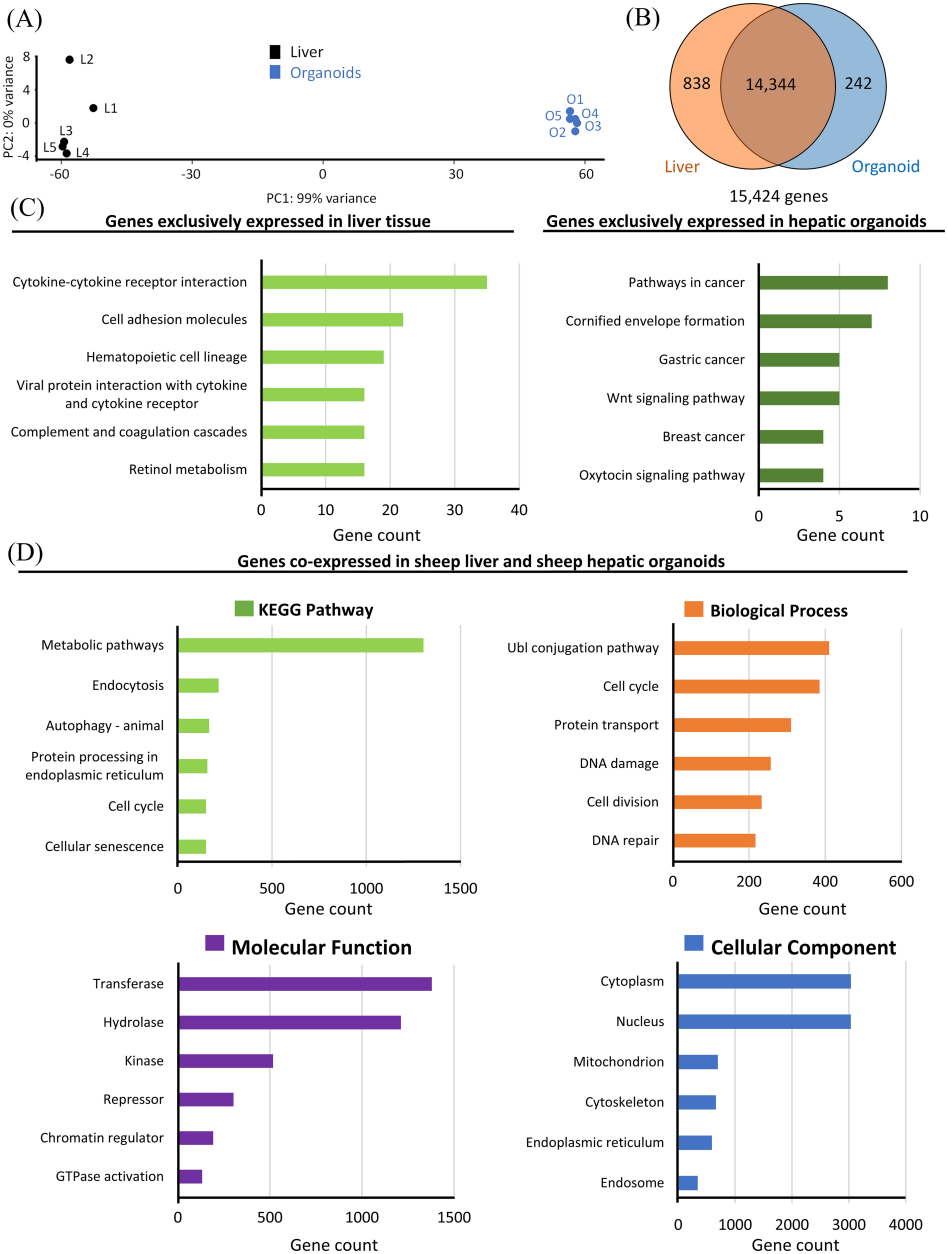
Transcriptional analysis of the sheep liver and hepatic organoids. **(A)** Principal component analysis including sheep liver samples (L1–L5, black dots) and sheep hepatic organoids (O1–O5, blue dots). **(B)** Venn diagram performed with the genes expressed in sheep liver tissue and sheep hepatic organoids (the orange circle includes the number of genes expressed in the liver tissue, whereas the blue circle includes the number of genes expressed in hepatic organoids). The number of genes co-expressed in both systems is included in the intersection of the two circles. **(C)** Functional enrichment analysis of the 838 gene transcripts exclusively detected in liver tissue (left panel) and the 242 gene transcripts exclusively detected in sheep hepatic organoids (right panel). The six most represented KEGG (Kyoto Encyclopedia of Genes and Genomes) pathways are represented as green bars. **(D)** Functional enrichment analysis of the 14,344 genes co-expressed in the liver tissue and hepatic organoids. The six most represented pathways in each category (KEGG pathway, biological process, cellular compartment, and molecular function) are represented as colored bars.

In order to compare gene expression profiles from sheep hepatic organoids and sheep liver tissue, RNA-seq analysis was carried out. Principal component analysis (Figure 3A) resulted in statistically significant clusters (99% confidence intervals) for each sample type. The analysis of the transcriptional profiles of hepatic organoids and liver tissue revealed that, among the 15,424 genes transcribed (Supplementary Table S1), 14,344 genes were co-expressed in both hepatic organoids and liver, 838 genes were transcribed only in the liver tissue, and 242 genes were exclusively expressed in hepatic organoids (Figure 3B).

Those genes exclusively expressed were grouped using the DAVID free software in the six most significant KEGG pathways (Figure 3C), indicating that in liver tissue, these pathways were related to classical hepatic metabolic and immune functions, including retinol metabolism, complement and coagulation cascades, and cytokine–cytokine receptor interaction. Additional enriched pathways such as hematopoietic cell lineage and cell adhesion molecules further reflect the complex cellular composition and immunological role of the liver *in vivo*. In contrast, hepatic organoids showed enrichment of pathways mainly associated with signaling, proliferation, and epithelial differentiation, including Wnt signaling pathway, oxytocin signaling pathway, and several KEGG cancer-related pathways, the latter suggesting highly proliferative cellular systems. The pathway “cornified envelope formation” was also significantly enriched, suggesting differences in epithelial differentiation programs.

To determine the six most significant metabolic pathways, based on the KEGG results, as well as biological, cellular, and molecular categories of the 14,344 genes co-expressed both in the liver tissue and the hepatic organoids, functional enrichment analysis was carried out using the DAVID free software (Figure 3D). Most of the co-expressed genes are involved in metabolic pathways and are related to ubiquitin conjugation, cell cycle, and protein transport, indicating shared programs of conservation of protein quality control and cellular homeostasis mechanisms, proliferation and protein homeostasis across both systems. The most active cellular sites are cytoplasm and nucleus, and transferases and hydrolases are highly abundant.

**Figure 4:**
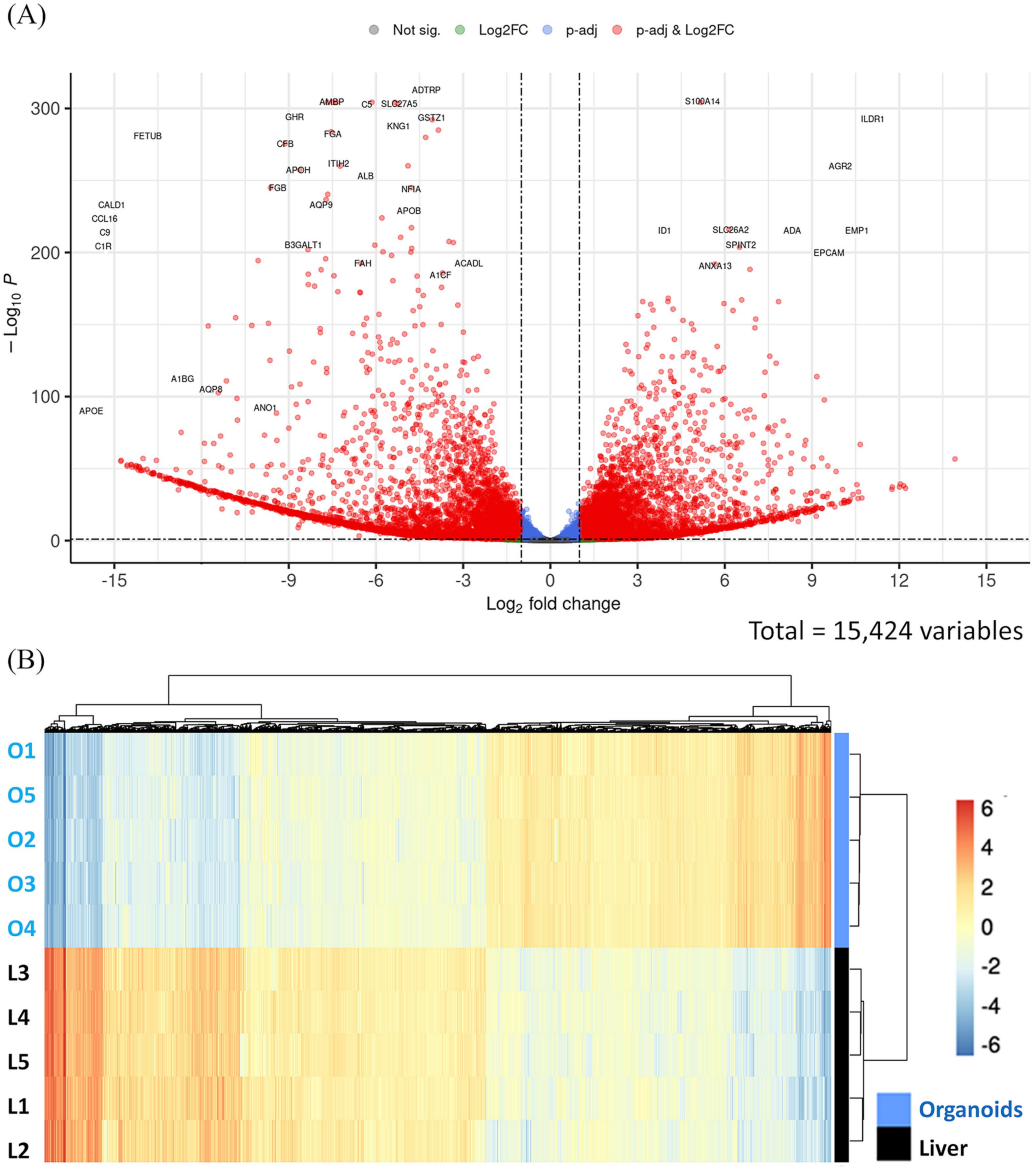
Differential expression between sheep liver and sheep hepatic organoids. **(A)** Volcano plot revealing the expression profile of significantly regulated genes. The horizontal axis represents the difference in expression, indicated as Log2 of the calculated fold change. The vertical axis represents the level of evidence, which is indicated as −log(P_adj_), with significantly regulated genes appearing at the top of the plot. Gray dots represent genes that show neither statistical significance nor differential expression. Blue dots indicate statistically significant genes without meaningful changes in expression. Green dots represent genes with expression changes that are not statistically significant. Red dots highlight genes that are both statistically significant and differentially expressed (downregulated genes in organoids on the left and upregulated genes in organoids on the right). **(B)** Heatmap showing the genes with the highest differential expression between sheep hepatic organoids (O1–O5) and sheep liver tissue (L1–L5). The applied filters are: Log2FC < −1 or >1, and *q*-value < 0.1. Color scale indicates gene expression levels from low (blue) to high (red). Dendrograms reflect hierarchical clustering based on sample similarity.

Differential expression analysis between liver tissue and hepatic organoids was carried out with DESeq2. A total of 3,974 genes were upregulated and 5,093 downregulated in hepatic organoids compared to the liver tissue. The Volcano plot (Figure 4A) reveals a good balance between overexpressed and repressed genes, indicating that the differential expression is significant. The genes with the highest differential expression between both study groups can be visualized with a heatmap (Figure 4B). The six most upregulated genes in hepatic organoids compared to liver tissue (Table 2) were related to enhanced proliferative, secretory, and membrane remodeling activities. On the other hand, several genes with established roles in mature liver function were significantly downregulated in the organoid condition (Table 3), including plasma glycoproteins, core components of the complement cascade, calcium-binding regulatory proteins involved in vitamin C biosynthesis, or fatty acid transporters (see Discussion).

**Table 2:** Top six most overexpressed genes in sheep hepatic organoids compared to liver tissue, ranked by *p*-value.

Gene product	Description^a^	Log2FC^b^	*p*-value
S100A14	S100 calcium binding protein A14. Predicted to enable calcium ion binding activity; calcium-dependent protein binding activity; and chemokine receptor binding activity. Predicted to be involved in positive regulation of leukocyte chemotaxis; response to lipopolysaccharide; and toll-like receptor 4 signaling pathway	5.2	0.0
SLC26A2	Solute carrier family 26 member 2. It enables solute:inorganic anion antiporter activity and sulfate transmembrane transporter activity. Involved in chondrocyte differentiation; chondrocyte proliferation; and sulfate transmembrane transport	6.1	1.3 × 10^−219^
SPINT2	Serine peptidase inhibitor, Kunitz type 2. Predicted to enable serine-type endopeptidase inhibitor activity. It acts upstream of or within basement membrane organization; chordate embryonic development; and establishment or maintenance of cell polarity	6.5	4.2 × 10^−207^
ANXA13	Annexin A13. Predicted to enable calcium ion binding activity and phospholipid binding activity	5.7	2.3 × 10^−195^
AGR2	Anterior gradient 2, protein disulfide isomerase family member. Predicted to enable dystroglycan binding activity; epidermal growth factor receptor binding activity; and protein homodimerization activity. Involved in digestive tract morphogenesis; mucus secretion; and positive regulation of developmental growth. Acts upstream of or within inflammatory response and lung goblet cell differentiation	6.9	1.4 × 10^−191^
PLP2	Proteolipid protein 2. Predicted to enable chemokine binding activity	4.1	1.8 × 10^−171^

a Information summarized from the National Library of Medicine (https://www.ncbi.nlm.nih.gov/datasets/gene/).

b Log2FC = Log2 of the calculated fold change.

**Table 3:** Top six most downregulated genes in sheep hepatic organoids compared to liver tissue, ranked by *p*-value.

Gene product	Description^a^	Log2FC^b^	*p*-value
AMBP	Alpha-1-microglobulin/bikunin precursor. This gene encodes a fusion protein that undergoes proteolytic processing to generate two mature proteins: alpha-1-microglobulin (A1m) is a heme-binding plasma glycoprotein of the lipocalin superfamily of proteins that bind to hydrophobic molecules, whereas bikunin belongs to the superfamily of Kunitz-type protease inhibitors	−7.5	0.0
C5	Complement C5	−6.1	0.0
ITIH1	Inter-alpha-trypsin inhibitor heavy chain 1. This gene encodes a heavy chain of inter-alpha trypsin inhibitor (IaI) family of plasma serine protease inhibitors	−7.4	0.0
LOC101110449	UDP-glucuronosyltransferase 3A1-like	−5.4	0.0
RGN	Regucalcin. It enables gluconolactonase activity. Involved in L-ascorbic acid biosynthetic process	−7.7	0.0
SLC27A5	Solute carrier family 27 member 5. It enables cholate-CoA ligase activity and fatty acid transmembrane transporter activity. It acts upstream of or within several processes, including ketone body biosynthetic process; long-chain fatty acid import across plasma membrane; and triglyceride mobilization	−5.2	2.5 × 10^−307^

a Information summarized from the National Library of Medicine (https://www.ncbi.nlm.nih.gov/datasets/gene/).

b Log2FC = Log2 of the calculated fold change.

**Figure 5:**
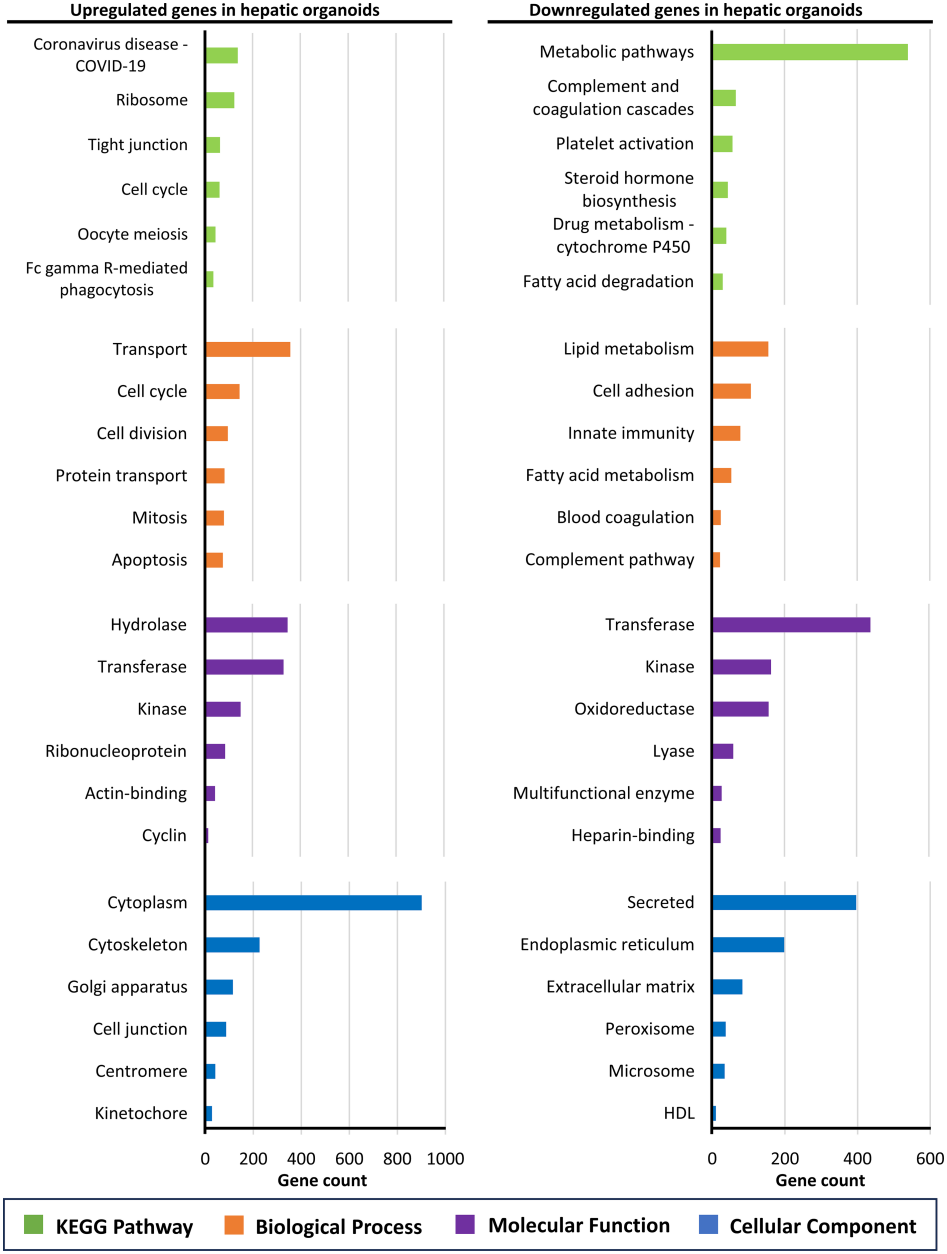
Functional enrichment analysis of the differentially expressed genes in sheep hepatic organoids compared to sheep liver tissue. The left panel shows the analysis of the 3,974 genes upregulated in hepatic organoids, whereas the right panel shows the analysis of the 5,093 downregulated genes in hepatic organoids. The six most represented pathways in each category (KEGG pathway, biological process, cellular compartment, and molecular function) are represented as colored bars.

A detailed characterization of the differences in the gene expression profile was performed by functional enrichment analysis of the 3,974 upregulated and 5,093 downregulated genes in hepatic organoids compared to the liver tissue using the DAVID server. The most significant metabolic pathways (up to six), based on the KEGG results, as well as gene ontology categories (biological process, cellular component and molecular function) are represented for the upregulated and downregulated genes (Figure 5). Overall, genes upregulated in organoids were predominantly associated with cell proliferation, protein transport, and structural organization. In contrast, genes downregulated in organoids were largely involved in metabolic processes, lipid and steroid metabolism, drug metabolism, extracellular matrix organization, and immune-related functions.

### 3.3 Response of ovine hepatic organoids to the addition methyl donors

**Figure 6:**
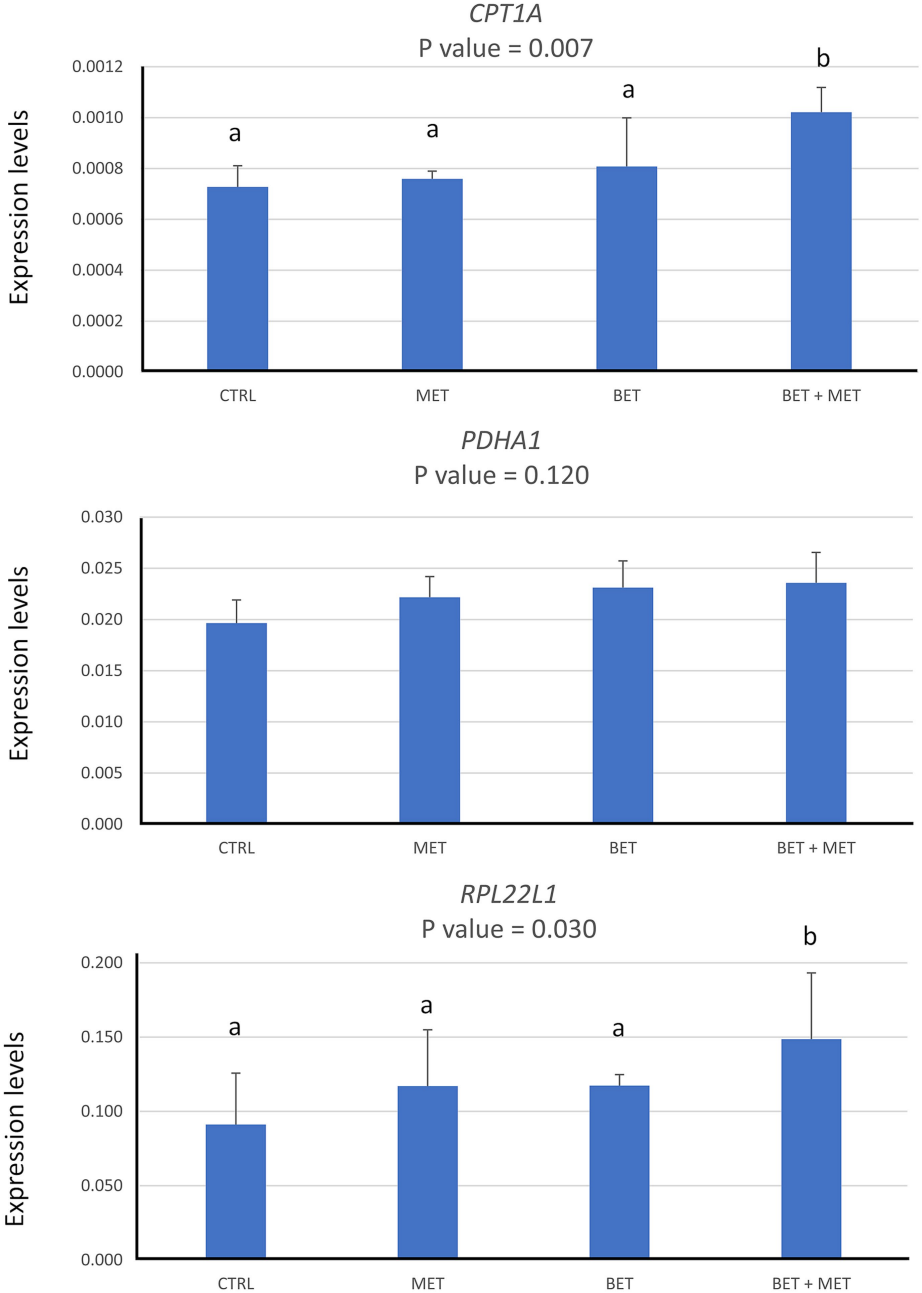
Expression of *CPT1A*, *PDH1A*, and *RPL22L1* genes observed by RT-qPCR. Data resulting from the addition of methionine (Met), betaine (Bet) or both compounds simultaneously (Met + Bet) were represented as the efficiency-corrected target quantity (N_0_) values. Different letters above the bars indicate significant differences among treatments (*p* < 0.05).

In order to validate the function of the 3D *in vitro* model for studying hepatic metabolism in ruminants, the potential effects of DL-methionine, betaine, and the combination of both compounds on the expression of key genes involved in hepatic metabolism and cell proliferation was evaluated *in vitro* using ovine hepatic organoids. According to the results observed in Figure 6, a significant increase (*p* < 0.05) in the expression of the *CPT1A* (liver-predominant carnitine palmitoyltransferase isoform 1A, representative of mitochondrial β-oxidation of fatty acids) and *RPL22L1* genes (ribosomal protein L22 like 1, representative of cellular proliferation) was observed in ovine hepatic organoids when DL-methionine and betaine were supplied together, whereas no clear effects on the expression of these genes was observed when these compounds were administered separately. No significant differences were found for the *PDHA1* gene (pyruvate dehydrogenase (lipoamide) alpha 1, representative of mitochondrial aerobic metabolism), with any of the treatments.

## 4 Discussion

The development of 3D systems in sheep represents a key advance for investigating physiological and pathological processes relevant to animal production and health, as they more accurately recapitulate tissue architecture, functionality, and cellular interactions observed *in vivo*. Compared with traditional 2D models, 3D systems provide an enhanced capacity to study nutrient absorption mechanisms, immune responses, host–pathogen interactions, and the effects of drugs or bioactive compounds, while also contributing to a reduction in the reliance on animal models (15, 19–22). In this context, the previous generation of sheep duodenum intestinal organoids and their implementation in high-throughput screening platforms (23) have demonstrated the feasibility and potential of ovine 3D models as robust tools for veterinary and translational research, paving the way for their application to other tissues and biological processes of interest. In this context, we have implemented the use of sheep hepatic organoids in metabolic studies. This development should also be considered within the broader expansion of organoid technologies in livestock species (13). In recent years, bovine and porcine organoid systems have been successfully established from intestinal and epithelial tissues, demonstrating their capacity to recapitulate key aspects of *in vivo* physiology, including nutrient transport, epithelial barrier function, and host–pathogen interactions (24). In cattle, intestinal organoids have been widely used as models to study digestive physiology and enteric infections, while also preserving tissue-specific gene expression patterns and functional responses (25, 26). Similarly, porcine organoids have emerged as robust models for investigating host–microbe interactions, nutritional development, drug discovery, and gene editing potential due to their close physiological resemblance to other monogastric and agricultural species (27). Collectively, these advances highlight the increasing relevance of livestock-derived organoids as physiologically meaningful *in vitro* systems for veterinary and animal production research. Within this context, the development of ovine hepatic organoids extends these efforts to a metabolically central organ in ruminant physiology, providing a complementary platform for studying liver-specific processes in a species-relevant manner.

The 3D structures developed in this work, generated from the liver of a male lamb, showed spherical morphology, early progressive growth, and preserved integrity after long-term storage, which is consistent with previous reports on hepatic organoids generated from other animals (15, 28). Staining with H&E showed that the organoids formed cystic epithelial structures, with most cells displaying morphological features consistent with hepatocytes. Additionally, ZO-1, a tight junction protein located at the apical boundary, was detected at the luminal face of these cells, reflecting an apical-in polarity similar to the organization of bile canaliculi *in vivo* (29). Cytoplasmic accumulation of albumin, and the presence of glycogen-loaded vacuoles further supports the metabolic resemblance of these cells to hepatocytes (30, 31). Furthermore, they were not present in all cells and their levels varied among individual cells, reflecting the functional heterogeneity characteristic of native hepatic tissue. Such variation is consistent with a mixed population of mature hepatocyte-like cells, hepatic progenitors, and possibly other hepatic cells. This is further supported by the partial stratification observed in some organoids, which may reflect active proliferation or heterogeneity in differentiation, and by the expression of certain markers in the organoid cells. As reported by the manufacturer, organoids maintained in HepatiCult™ Mouse Organoid Growth Medium develop an epithelial compartment that expresses markers associated with hepatic stem and progenitor cells (*AXIN2*, *SOX9*, and *CD44*), biliary duct cells (*KRT19* and *HNF1b*), and hepatocytes (*HNF4a* and *AFP*). The expression of these markers, together with additional genes characteristic of the intrahepatic bile duct, such as *CFTR* (32) (Supplementary Table S1), support the presence of a heterogeneous cell population in the organoids developed in this work. This cellular diversity resembles the *in vivo* hepatic microenvironment and is coincident with previous reports describing organotypic cultures derived from adult tissue (15, 33, 34). One limitation of the current study is that hepatic organoids were derived from a single animal. Although the results demonstrate the feasibility and reproducibility of the system under the conditions tested, biological variability between animals cannot be assessed. Future studies will include organoids derived from multiple individuals of different ages or physiological states to evaluate inter-animal variability and to strengthen the robustness and generalizability of the model.

Transcriptomics analysis highlighted conserved mechanisms coordinating cell cycle progression with protein turnover and trafficking, supporting the physiological relevance of hepatic organoids as a model system. However, comparison of the transcriptional profiles of liver tissue and hepatic organoids revealed that the *in vitro* 3D system only partially recapitulates the metabolic and signaling pathways typical of mature liver, retaining transcriptional programs consistent with a more immature or actively remodeling cellular state compared to native liver tissue. This phenomenon is a common feature of hepatic organoids, which display transcriptional signatures resembling fetal or neonatal hepatocytes (35). In the present study, culture conditions were primarily optimized for organoid expansion and maintenance rather than for inducing full hepatocyte maturation. This likely contributes to the transcriptional profile observed, which reflects a proliferative and developmentally immature state compared to adult liver tissue. Therefore, future work should focus on optimizing culture conditions to promote hepatocyte maturation, including modulation or withdrawal of proliferative signals, incorporation of differentiation-inducing factors, or the use of co-culture systems with non-parenchymal liver cells. Such approaches may enhance the physiological relevance of the model and improve its capacity to recapitulate adult liver metabolic functions.

The analysis of the six most up- and downregulated genes in hepatic organoids compared with hepatic tissue provided key insights into the characterization of the physiology of this ovine 3D system. The coordinated overexpression of *S100A14*, *SLC26A2*, *SPINT2*, *ANXA13*, *AGR2*, and *PLP2* in hepatic organoids reflects the developmental, proliferative, and remodeling state of the organoids rather than the phenotype of differentiated liver tissue. The *S100* genes play an important role in tumorigenesis by regulating cell proliferation, invasion, metastasis, cell survival, or cell death (36). Namely, *S100A14* encodes a calcium-binding protein implicated in the regulation of cell proliferation and survival via signaling pathways such as RAGE-ERK/NF-κB in epithelial cells (37), consistent with enhanced proliferative programs in organoids. The increased expression of *SLC26A2* in hepatic organoids compared with native ovine liver likely reflects the immature and proliferative state of organoid cells rather than a liver-specific function. *SLC26A2* encodes a sulfate transporter required for intracellular sulfate supply and subsequent proteoglycan sulfation, a process essential for extracellular matrix biosynthesis and growth factor signaling during development and tissue remodeling. Although classically associated with cartilage disorders, *SLC26A2* is expressed in multiple non-cartilaginous tissues, supporting a broader role in sulfate homeostasis across diverse cell types (38). Its upregulation in organoids is therefore consistent with enhanced biosynthetic and matrix-related demands characteristic of development-like or regenerating systems rather than terminally differentiated native liver tissue. *SPINT2* encodes the Kunitz-type serine protease inhibitor HAI-2, which modulates pericellular proteolysis and epithelial integrity and inhibits the hepatocyte growth factor activator, thus modulating proliferation and protecting epithelial integrity during tissue remodeling (39, 40), thereby likely protecting the structure and integrity of the organoid. *ANXA13* is a member of the annexin family that binds phospholipids in a calcium-dependent manner and is associated with membrane dynamics and growth regulation in proliferative epithelial cells (41), thus playing a role in organoid proliferation and differentiation. *AGR2* encodes an endoplasmic reticulum-resident protein disulfide isomerase that facilitates protein folding and secretion, supporting high biosynthetic demand in development and regeneration (42). Finally, *PLP2* encodes an integral membrane protein of the endoplasmic reticulum (ER). Knockdown studies show that reduced *PLP2* increases ER stress and apoptosis (43), while overexpression correlates with enhanced proliferation and modulation of cell migration (44, 45). Thus, *PLP2* upregulation in organoids likely reflects the high biosynthetic activity, ER stress management, and proliferative demands in these immature tissue-like structures and may facilitate cell survival, structural integrity, and adaptive responses to high metabolic and remodeling activity.

On the other hand, the consistent downregulation of *AMBP*, *C5*, *ITIH1*, *RGN*, and *SLC27A5* in hepatic organoids compared with mature liver suggests that these structures have not fully activated transcriptional programs associated with mature hepatocyte phenotype and systemic liver functions. *AMBP* encodes a precursor of α1-microglobulin and bikunin that is highly expressed in liver and regulated by hepatocyte nuclear factors, reflecting its role in hepatocyte-specific secretory activity and antioxidative protection *in vivo*, as it is regulated by liver-enriched transcription factors (46). *C5* encodes a central complement component involved in innate immunity and systemic inflammatory responses (47, 48) and its downregulation is expected in a tissue-restricted organoid model lacking systemic immune interactions. *ITIH1*, part of the inter-alpha-trypsin inhibitor family that contributes to extracellular matrix stability and circulatory protease inhibition, is also principally expressed in liver, and its repression is consistent with reduced mature hepatocyte secretory capacity (49). *RGN* (regucalcin) is a calcium-binding regulatory protein with preferential liver expression that contributes to intracellular calcium signaling and homeostasis, functions more prominent in terminally differentiated hepatocytes than in proliferative progenitor-like cells (50) and is involved in vitamin C biosynthesis (51). Finally, *SLC27A5* encodes a liver-specific bile acid and long-chain fatty acid transport enzyme (*FATP5*) involved in lipid metabolism and bile acid conjugation (52, 53); its repression aligns with the lower metabolic and lipid processing activity of organoids relative to adult liver tissue. Collectively, these patterns suggest that organoids preferentially express genes associated with developmental and proliferative states, while genes defining mature hepatic metabolism, immune-related secretion, and systemic homeostasis are comparatively suppressed, thus supporting an interpretation of organoids as developmentally immature, proliferative structures with enhanced matrix remodeling and cellular homeostatic programs, distinct from mature liver biology.

This scenario is confirmed by the functional enrichment analysis, which highlights marked transcriptomic differences between hepatic organoids and native liver tissue. The upregulation of genes involved in the cell cycle, mitosis, and protein transport suggests high proliferative and biosynthetic activity in organoids. Enrichment of terms related to cell junctions and cytoskeleton reflects structural integrity and dynamic cellular organization, characteristic of growing organoids (54, 55). It should be noted that the enrichment of the “Coronavirus disease – COVID-19” KEGG pathway does not indicate viral infection in the organoids. Most of the upregulated genes in this category (approximately 80%) encode ribosomal proteins, while the remaining genes are involved in intracellular signaling pathways (*MAPK*, *NF-κB*, *PI3K*), both representing fundamental cellular processes that are highly active in proliferating cells (56–58). Thus, the mapping of these genes to the COVID-19 pathway reflects their functional overlap with processes exploited by the virus, rather than the presence of SARS-CoV-2 in the system. Conversely, the downregulation of genes associated with classical hepatic metabolic pathways, including lipid metabolism, steroid biosynthesis, and cytochrome P450-mediated drug metabolism (59, 60), may indicate that organoids have incomplete functional maturation compared to adult liver tissue. Reduced expression of genes related to the extracellular matrix and secreted components may reflect differences in tissue architecture and the absence of complex cell–matrix interactions *in vitro*. Additionally, decreased expression of genes associated with innate immune response and complement cascades is expected, as the sheep hepatic organoids developed in this work lack resident immune cells found in the liver. Most current liver organoid systems lack immune cells, such as tissue-resident macrophages, limiting their ability to recapitulate the complex immunological functions of the liver *in vivo* (61). Overall, these findings emphasize the potential of hepatic organoids as models to study cell proliferation and basic liver biology, while highlighting the need for further optimization to improve functional maturation and better mimic adult liver physiology.

Finally, in order to validate the applicability of this 3D model for hepatic metabolism assays, we assessed the effects of methionine and betaine supplementation on the expression of two genes involved in general metabolic pathways (*CPT1A* and *PDHA1*) and one gene related to cell proliferation (*RPL22L1*). Methionine is a sulfur-containing essential amino acid and one of the most limiting amino acid in ruminant diets. It plays a role not only in protein synthesis, but also as an important methyl donor that can be converted into S-adenosylmethionine and homocysteine (62). Specifically, appropriate supplementation with methionine or S-adenosylmethionine has been shown to provide a wide range of health-promoting effects, to be noted the amelioration of hepatic steatosis (63, 64). However, elevated homocysteine levels can increase oxidative stress and inflammation, particularly at hepatic level (65, 66). This is the reason why recent studies have shown that methionine restriction may also be positive in terms of alleviating hepatic steatosis and achieving other health benefits (67–69). Therefore, it is pertinent searching for strategies to optimize methionine as a feed additive. In this sense, betaine, a choline derivative, is important because of its role in the donation of methyl groups to homocysteine to form methionine (70). Consequently, the combination of methionine and betaine in the diet of animals might provide a synergy between both compounds, thus allowing to benefit from the positive effects of methionine supplementation without being impaired by high homocysteine levels. According to the results obtained in the present study, the addition of either DL-methionine or betaine to the sheep hepatic organoids, did not provide significant differences in the expression of any of the three genes tested. Previous *in vitro* results in bovine hepatocytes supplemented with different concentrations of DL-methionine showed that *CPT1A* expression was not affected after 24 h of treatment (71), thus confirming our results with methionine. Interestingly, dietary supplementation of betaine in the feed supplied to lying hens gave rise to the activation of *CPT1A* gene expression in the liver, thus promoting fatty acid β-oxidation (72). In our 3D *in vitro* model, the combined action of both DL-methionine and betaine gave rise to an increased expression of the gene encoding *CPT1A*, which is the liver-predominant isoform of carnitine palmitoyltransferase 1. This is a key enzyme controlling the rate-limiting step of mitochondrial β-oxidation of long-chain fatty acids (73), and its increased expression may indicate a shift toward greater fatty acid oxidation in response to augmented methyl donor availability, or to increased methionine availability promoted by the betaine recycling of homocysteine. The same mechanisms may be also valid to explain the significant upregulation of the *RPL22L1* gene (encoding a ribosomal protein homologous to L22). This gene has been shown to participate in cell proliferation and tumor progression through modulation of signaling pathways (74), so its upregulation suggests enhanced protein synthesis and cellular growth, consistent with the role of methionine and betaine in one-carbon metabolism and methylation reactions (75).

These results suggest a potential shift toward enhanced mitochondrial β-oxidation of fatty acids and cellular proliferation when DL-methionine and betaine are supplied together, thus supporting a potential synergistic effect of both compounds, although this remains to be functionally validated. In fact, this is a key limitation of this study, since metabolic activity was inferred from gene expression data rather than direct functional assays. Therefore, future studies should incorporate functional approaches, such as measurements of mitochondrial respiration, β-oxidation rates, or metabolite profiling, to validate the metabolic capacity of ovine hepatic organoids.

In summary, sheep hepatic organoids represent a promising and physiologically relevant 3D model to study liver biology, capturing key structural and transcriptional features while retaining a proliferative and developmentally immature phenotype compared to adult liver tissue. Although further optimization is required to enhance functional maturation and fully recapitulate metabolic activity, this model provides a valuable platform for exploring cellular processes and metabolic regulation *in vitro*, thereby highlighting the utility of ovine 3D hepatic models for investigating nutrient-gene interactions in ruminants before being tested *in vivo*.

## Data Availability

The data presented in the study are deposited in the DIGITAL.CSIC repository, accesion number 418054 (http://hdl.handle.net/10261/418054).

## References

[1] Trefts E Gannon M Wasserman DH. The liver. Curr Biol. (2017) 27:R1147–51. doi: 10.1016/j.cub.2017.09.019 29112863 PMC5897118

[2] Reynolds CK. Economics of visceral energy metabolism in ruminants: tool keeping or internal revenue service? J Anim Sci. (2002) 80:E74–84. doi: 10.2527/animalsci2002.80E-Suppl_2E74x

[3] Hanigan MD. Quantitative aspects of ruminant splanchnic metabolism as related to predicting animal performance. Anim Sci. (2005) 80:23–32. doi: 10.1079/ASC40920023

[4] Armentano LE. Ruminant hepatic metabolism of volatile fatty acids, lactate and pyruvate. J Nutr. (1992) 122:838–42. doi: 10.1093/jn/122.suppl_3.838, 1542055

[5] Tan P Liu H Zhao J Gu X Wei X Zhang X . Amino acids metabolism by rumen microorganisms: nutrition and ecology strategies to reduce nitrogen emissions from the inside to the outside. Sci Total Environ. (2021) 800:149596. doi: 10.1016/j.scitotenv.2021.149596, 34426337

[6] Beigh YA Ganai AM Ahmad HA. Prospects of complete feed system in ruminant feeding: a review. Vet World. (2017) 10:424–37. doi: 10.14202/vetworld.2017.424-437, 28507415 PMC5422247

[7] Saxton SH Stevens KR. 2D and 3D liver models. J Hepatol. (2023) 78:873–5. doi: 10.1016/j.jhep.2022.06.022 36038394

[8] Dall M Stocks B Cervone DT Deshmukh AS Treebak JT. Hepatocyte dedifferentiation in 2D culture reveals extensive transcriptomic and proteomic rewiring. Hepatol Commun. (2025) 9:e0795. doi: 10.1097/HC9.0000000000000795, 41111196 PMC12506984

[9] Huch M Gehart H van Boxtel R Hamer K Blokzijl F Verstegen MM . Long-term culture of genome-stable bipotent stem cells from adult human liver. Cell. (2015) 160:299–312. doi: 10.1016/j.cell.2014.11.050, 25533785 PMC4313365

[10] Luce E Messina A Duclos-Vallée JC. Hepatic organoids as a platform for liver disease modeling and the development of novel therapies. Clin Res Hepatol Gastroenterol. (2025) 49:102647. doi: 10.1016/j.clinre.2025.102647 40615111

[11] Brooks A Liang X Zhang Y Zhao CX Roberts MS Wang H . Liver organoid as a 3D *in vitro* model for drug validation and toxicity assessment. Pharmacol Res. (2021) 169:105608. doi: 10.1016/j.phrs.2021.105608, 33852961

[12] Iqbal W Wang Y Sun P Zhou X. Modeling liver development and disease in a dish. Int J Mol Sci. (2023) 24:15921. doi: 10.3390/ijms242115921, 37958904 PMC10650907

[13] Kwon DH Kwon H Jang G. Organoid-based platforms in livestock: current advances and future prospects. Res Vet Sci. (2026) 198:105985. doi: 10.1016/j.rvsc.2025.105985, 41314120

[14] Vinyard JR Faciola AP. Unraveling the pros and cons of various *in vitro* methodologies for ruminant nutrition: a review. Transl Anim Sci. (2022) 6:txac130. doi: 10.1093/tas/txac130 36213308 PMC9536435

[15] González-Montero MC Andrés-Rodríguez J Criado M Andrés S Galli G Fernández-Rubio C . Development of a high-throughput screening platform and a pathogenesis model for Leishmania infection based on mouse hepatic organoids. Int J Mol Sci. (2025) 26:12180. doi: 10.3390/ijms262412180, 41465604 PMC12734288

[16] Fujii E Yamazaki M Kawai S Ohtani Y Watanabe T Kato A . A simple method for histopathological evaluation of organoids. J Toxicol Pathol. (2018) 31:81–5. doi: 10.1293/TOX.2017-0060 29479145 PMC5820108

[17] Huang DW Sherman BT Lempicki RA. Systematic and integrative analysis of large gene lists using DAVID bioinformatics resources. Nat Protoc. (2009) 4:44–57. doi: 10.1038/nprot.2008.211, 19131956

[18] Sherman BT Hao M Qiu J Jiao X Baseler MW Lane HC . DAVID: a web server for functional enrichment analysis and functional annotation of gene lists (2021 update). Nucleic Acids Res. (2022) 50:W216–21. doi: 10.1093/nar/gkac194 35325185 PMC9252805

[19] Zietek T Giesbertz P Ewers M Reichart F Weinmüller M Urbauer E . Organoids to study intestinal nutrient transport, drug uptake and metabolism - update to the human model and expansion of applications. Front Bioeng Biotechnol. (2020) 8:577656. doi: 10.3389/fbioe.2020.577656, 33015026 PMC7516017

[20] Gopallawa I Gupta C Jawa R Cyril A Jawa V Chirmule N . Applications of organoids in advancing drug discovery and development. J Pharm Sci. (2024) 113:2659. doi: 10.1016/j.xphs.2024.06.016, 39002723

[21] Dehnavi M Galli G García-Estrada C Balaña-Fouce R Giráldez FJ Alonso M . An ovine intestinal organoid-macrophage co-culture model to test the effects of ovine colostrum exosomes on intestinal barrier function and inflammation. Int J Mol Sci. (2025) 26:11406. doi: 10.3390/ijms262311406, 41373563 PMC12692019

[22] Gao Q Chow SK Shinohara I Murayama M Susuki Y Morita M . Can alternatives to animal testing yield useful information regarding biological mechanisms and drug discovery? J Orthop Translat. (2025) 55:132. doi: 10.1016/j.jot.2025.08.005, 41542102 PMC12799505

[23] Galli G Melcón-Fernández E de Garnica García MG Martínez-Fernández B Dehnavi M Andrés S . Development of sheep duodenum intestinal organoids and implementation of high-throughput screening platform for veterinary applications. Int J Mol Sci. (2025) 26:3452. doi: 10.3390/ijms26073452, 40244396 PMC11989482

[24] Beaumont M Blanc F Cherbuy C Egidy G Giuffra E Lacroix-Lamandé S . Intestinal organoids in farm animals. Vet Res. (2021) 52:33. doi: 10.1186/s13567-021-00909-x, 33632315 PMC7905770

[25] Hamilton CA Young R Jayaraman S Sehgal A Paxton E Thomson S . Development of *in vitro* enteroids derived from bovine small intestinal crypts. Vet Res. (2018) 49:54. doi: 10.1186/s13567-018-0547-5, 29970174 PMC6029049

[26] Kawasaki M Dykstra GD McConnel CS Burbick CR Ambrosini YM. Adult bovine-derived small and large intestinal organoids: *in vitro* development and maintenance. J Tissue Eng Regen Med. (2023):3095002:2023. doi: 10.1155/2023/3095002 38873240 PMC11175594

[27] Ma P Fang P Ren T Fang L Xiao S. Porcine intestinal organoids: overview of the state of the art. Viruses. (2022) 14:1110. doi: 10.3390/v14051110, 35632851 PMC9147602

[28] Hu H Gehart H Artegiani B López-Iglesias C Dekkers F Basak O . Long-term expansion of functional mouse and human hepatocytes as 3D organoids. Cell. (2018) 175:1591. doi: 10.1016/j.cell.2018.11.013, 30500538

[29] Fiorotto R Mariotti V Taleb SA Zehra SA Nguyen M Amenduni M . Cell-matrix interactions control biliary organoid polarity, architecture, and differentiation. Hepatol Commun. (2023) 7:e0094. doi: 10.1097/HC9.0000000000000094, 36972396 PMC10503667

[30] Ramli MNB Lim YS Koe CT Demircioglu D Tng W Gonzales KAU . Human pluripotent stem cell-derived organoids as models of liver disease. Gastroenterology. (2020) 159:1471. doi: 10.1053/j.gastro.2020.06.010, 32553762

[31] Pérez-García A Hurtado-Carneiro V Herrero-De-Dios C Dongil P García-Mauriño JE Sánchez MD . Storage and utilization of glycogen by mouse liver during adaptation to nutritional changes are Glp-1 and Pask dependent. Nutrients. (2021) 13:2552. doi: 10.3390/nu13082552, 34444712 PMC8399311

[32] Cohn JA Strong TV Picciotto MR Nairn AC Collins FS Fitz JG. Localization of the cystic fibrosis transmembrane conductance regulator in human bile-duct epithelial cells. Gastroenterology. (1993) 105:1857. doi: 10.1016/0016-5085(93)91085-V, 7504645

[33] Messner S Fredriksson L Lauschke VM Roessger K Escher C Bober M . Transcriptomic, proteomic, and functional long-term characterization of multicellular three-dimensional human liver microtissues. Appl Vitr Toxicol. (2018) 4:1. doi: 10.1089/aivt.2017.0022 PMC7500040 32953943

[34] Ariño S Ferrer-Lorente R Serrano G Zanatto L de la Martínez-García Torre RA Gratacós-Ginès J . Patient-derived liver organoids recapitulate liver epithelial heterogeneity and enable precision modeling of alcohol-related liver disease. J Hepatol. (2025) 84:135. doi: 10.1016/j.jhep.2025.07.014 40754225

[35] Sun XC Kong DF Zhao J Faber KN Xia Q He K. Liver organoids: established tools for disease modeling and drug development. Hepatol Commun. (2023) 7:e0105. doi: 10.1097/HC9.0000000000000105, 36972388 PMC10043560

[36] Jiang H Hu H Tong X Jiang Q Zhu H Zhang S. Calcium-binding protein S100P and cancer: mechanisms and clinical relevance. J Cancer Res Clin Oncol. (2012) 138:1–9. doi: 10.1007/s00432-011-1062-5, 21947242 PMC11824467

[37] Jin Q Chen H Luo A Ding F Liu Z. S100A14 stimulates cell proliferation and induces cell apoptosis at different concentrations via receptor for advanced glycation end products (RAGE). PLoS One. (2011) 6:e19375. doi: 10.1371/journal.pone.0019375, 21559403 PMC3084824

[38] Haila S Hästbacka J Böhling T Karjalainen-Lindsberg ML Kere J Saarialho-Kere U. SLC26A2 (diastrophic dysplasia sulfate transporter) is expressed in developing and mature cartilage but also in other tissues and cell types. J Histochem Cytochem. (2001) 49:973. doi: 10.1177/002215540104900805, 11457925

[39] Szabo R Hobson JP Christoph K Kosa P List K Bugge TH. Regulation of cell surface protease matriptase by HAI2 is essential for placental development, neural tube closure and embryonic survival in mice. Development. (2009) 136:2653–63. doi: 10.1242/dev.038430, 19592578 PMC2709071

[40] Roversi FM Saad STO Machado-Neto JA. Serine peptidase inhibitor Kunitz type 2 (SPINT2) in cancer development and progression. Biomed Pharmacother. (2018) 101:278. doi: 10.1016/j.biopha.2018.02.100, 29499401

[41] McCulloch KM Yamakawa I Shifrin DA McConnell JRE Foegeding NJ Singh PK . An alternative N-terminal fold of the intestine-specific annexin A13a induces dimerization and regulates membrane-binding. J Biol Chem. (2019) 294:3454. doi: 10.1074/jbc.RA118.004571, 30610115 PMC6416438

[42] Delom F Mohtar MA Hupp T Fessart D. The anterior gradient-2 interactome. Am J Physiol Cell Physiol. (2020) 318:C40. doi: 10.1152/ajpcell.00532.2018 31644305

[43] Feng Z Zhou W Wang J Qi Q Han M Kong Y . Reduced expression of proteolipid protein 2 increases ER stress-induced apoptosis and autophagy in glioblastoma. J Cell Mol Med. (2019) 24:2847. doi: 10.1111/jcmm.14840, 31778016 PMC7077595

[44] Chen YH Hueng DY Tsai WC. Proteolipid protein 2 overexpression indicates aggressive tumor behavior and adverse prognosis in human gliomas. Int J Mol Sci. (2018) 19:3353. doi: 10.3390/ijms19113353, 30373180 PMC6274732

[45] Ghosh D Dutta A Kashyap A Upmanyu N Datta S. PLP2 drives collective cell migration via ZO-1-mediated cytoskeletal remodeling at the leading edge in human colorectal cancer cells. J Cell Sci. (2021) 134:jcs253468. doi: 10.1242/jcs.253468 34409455

[46] Rouet P Raguenez G Tronche F Mfou'ou V Salier JP. Hierarchy and positive/negative interplays of the hepatocyte nuclear factors HNF-1, −3 and −4 in the liver-specific enhancer for the human alpha-1-microglobulin/bikunin precursor. Nucleic Acids Res. (1995) 23:395–404. doi: 10.1093/nar/23.3.395, 7533900 PMC306689

[47] Mathern DR Heeger PS. Molecules great and small: the complement system. Clin J Am Soc Nephrol. (2015) 10:1636–50. doi: 10.2215/CJN.06230614, 25568220 PMC4559511

[48] Lubbers R van Essen MF van Kooten C Trouw LA. Production of complement components by cells of the immune system. Clin Exp Immunol. (2017) 188:183–94. doi: 10.1111/cei.12952, 28249350 PMC5383442

[49] Hamm A Veeck J Bektas N Wild PJ Hartmann A Heindrichs U . Frequent expression loss of inter-alpha-trypsin inhibitor heavy chain (ITIH) genes in multiple human solid tumors: a systematic expression analysis. BMC Cancer. (2008) 8:25. doi: 10.1186/1471-2407-8-25, 18226209 PMC2268946

[50] Yamaguchi M. Role of regucalcin in calcium signaling. Life Sci. (2000) 66:1769. doi: 10.1016/s0024-3205(99)00602-5 10809175

[51] Duque P Vieira CP Bastos B Vieira J. The evolution of vitamin C biosynthesis and transport in animals. BMC Ecol Evol. (2022) 22:84. doi: 10.1186/s12862-022-02040-7, 35752765 PMC9233358

[52] Doege H Baillie RA Ortegon AM Tsang B Wu Q Punreddy S . Targeted deletion of FATP5 reveals multiple functions in liver metabolism: alterations in hepatic lipid homeostasis. Gastroenterology. (2006) 130:1245–58. doi: 10.1053/j.gastro.2006.02.006 16618416

[53] Hubbard B Doege H Punreddy S Wu H Huang X Kaushik VK . Mice deleted for fatty acid transport protein 5 have defective bile acid conjugation and are protected from obesity. Gastroenterology. (2006) 130:1259–69. doi: 10.1053/j.gastro.2006.02.012, 16618417

[54] Morizane R Lamers MM. Organoids in disease modeling and regenerative medicine. Cell Mol Life Sci. (2025) 82:169. doi: 10.1007/s00018-025-05692-y, 40257505 PMC12011692

[55] Moss SP Bakirci E Feinberg AW. Engineering the 3D structure of organoids. Stem Cell Rep. (2025) 20:102379. doi: 10.1016/j.stemcr.2024.11.009, 39706178 PMC11784486

[56] Lu Y Wang S Jiao Y. The effects of deregulated ribosomal biogenesis in cancer. Biomolecules. (2023) 13:1593. doi: 10.3390/biom13111593, 38002277 PMC10669593

[57] Zhang W Liu HT. MAPK signal pathways in the regulation of cell proliferation in mammalian cells. Cell Res. (2002) 12:9–18. doi: 10.1038/sj.cr.7290105 11942415

[58] Shankar E Weis MC Avva J Shukla S Shukla M Sreenath SN . Complex systems biology approach in connecting PI3K-Akt and NF-κB pathways in prostate cancer. Cells. (2019) 8:201. doi: 10.3390/cells8030201, 30813597 PMC6468646

[59] Rui L. Energy metabolism in the liver. Compr Physiol. (2014) 4:177–97. doi: 10.1002/cphy.c130024, 24692138 PMC4050641

[60] Abdelmonem BH Abdelaal NM Anwer EKE Rashwan AA Hussein MA Ahmed YF . Decoding the role of CYP450 enzymes in metabolism and disease: a comprehensive review. Biomedicine. (2024) 12:1467. doi: 10.3390/biomedicines12071467, 39062040 PMC11275228

[61] Rezvani M. Human liver immunology: from *in vitro* models to new insights. Cell Mol Immunol. (2025) 22:1226–36. doi: 10.1038/s41423-025-01312-8 40604306 PMC12479813

[62] Elango R. Methionine nutrition and metabolism: insights from animal studies to inform human nutrition. J Nutr. (2020) 150:2518S–23S. doi: 10.1093/jn/nxaa155 33000159

[63] Vergani L Baldini F Khalil M Voci A Putignano P Miraglia N. New perspectives of S-Adenosylmethionine (SAMe) applications to attenuate fatty acid-induced steatosis and oxidative stress in hepatic and endothelial cells. Molecules. (2020) 25:4237. doi: 10.3390/molecules25184237 32942773 PMC7570632

[64] Navik U Sheth VG Sharma N Tikoo K. L-methionine supplementation attenuates high-fat fructose diet-induced non-alcoholic steatohepatitis by modulating lipid metabolism, fibrosis, and inflammation in rats. Food Funct. (2022) 13:4941–53. doi: 10.1039/d1fo03403k 35437549

[65] Matté C Stefanello FM Mackedanz V Pederzolli CD Lamers ML Dutra-Filho CS . Homocysteine induces oxidative stress, inflammatory infiltration, fibrosis and reduces glycogen/glycoprotein content in liver of rats. Int J Dev Neurosci. (2009) 27:337–44. doi: 10.1016/j.ijdevneu.2009.03.005, 19460627

[66] Stangl GI Schwarz FJ Jahn B Kirchgessner M. Cobalt-deficiency-induced hyperhomocysteinaemia and oxidative status of cattle. Br J Nutr. (2000) 83:3–6. doi: 10.1017/s0007114500000027, 10703458

[67] Yang Y Wang Y Sun J Zhang J Guo H Shi Y . Dietary methionine restriction reduces hepatic steatosis and oxidative stress in high-fat-fed mice by promoting H2S production. Food Funct. (2019) 10:61–77. doi: 10.1039/c8fo01629a 30534793

[68] Sun F Cao Y Cai C Li S Yu C Yao J. Regulation of nutritional metabolism in transition dairy cows: energy homeostasis and health in response to post-ruminal choline and methionine. PLoS One. (2016) 11:e0160659. doi: 10.1371/journal.pone.0160659 27501393 PMC4976856

[69] Liu R Diao Q Cui K. Effect of dietary methionine deficiency followed by a re-feeding phase on the hepatic antioxidant activities of lambs. Animals. (2020) 11:7. doi: 10.3390/ani11010007, 33374518 PMC7822206

[70] Bortz J Obeid R. The shuttling of methyl groups between folate and choline pathways. Nutrients. (2025) 17:2495. doi: 10.3390/nu17152495 40806080 PMC12348172

[71] Zhang Q Bertics SJ Luchini ND White HM. The effect of increasing concentrations of dl-methionine and 2-hydroxy-4-(methylthio) butanoic acid on hepatic genes controlling methionine regeneration and gluconeogenesis. J Dairy Sci. (2016) 99:8451–60. doi: 10.3168/jds.2016-11312, 27474977

[72] Yang Y Jin X Lu D Wu Y Meng F Zhang M . Betaine decreases hepatic lipid deposition through DNA 5 mC and RNA m6A methylation-mediated regulation of fatty acid metabolic genes expression in laying hens. Poult Sci. (2025) 104:105496. doi: 10.1016/j.psj.2025.105496, 40592294 PMC12269568

[73] Schlaepfer IR Joshi M. CPT1A-mediated fat oxidation, mechanisms, and therapeutic potential. Endocrinology. (2020) 161:bqz046. doi: 10.1210/endocr/bqz046, 31900483

[74] Yi X Zhang C Liu B Gao G Tang Y Lu Y . Ribosomal protein L22-like1 promotes prostate cancer progression by activating PI3K/Akt/mTOR signalling pathway. J Cell Mol Med. (2023) 27:403–11. doi: 10.1111/jcmm.17663, 36625246 PMC9889667

[75] Friso S Udali S De Santis D Choi SW. One-carbon metabolism and epigenetics. Mol Asp Med. (2017) 54:28–36. doi: 10.1016/j.mam.2016.11.007 27876555

